# Identification of Novel Interspersed DNA Repetitive Elements in the *Trypanosoma cruzi* Genome Associated with the 3′UTRs of Surface Multigenic Families

**DOI:** 10.3390/genes11101235

**Published:** 2020-10-21

**Authors:** Simone Guedes Calderano, Milton Yutaka Nishiyama Junior, Marjorie Marini, Nathan de Oliveira Nunes, Marcelo da Silva Reis, José Salvatore Leister Patané, José Franco da Silveira, Julia Pinheiro Chagas da Cunha, Maria Carolina Elias

**Affiliations:** 1 Laboratório de Parasitologia, Instituto Butantan, São Paulo 05503-900, Brazil; 2 Center of Toxins, Immune Response and Cell Signaling (CeTICS), Instituto Butantan, São Paulo 05503-900, Brazil; milton.nishiyama@butantan.gov.br (M.Y.N.J.); nathannunes@usp.br (N.d.O.N.); marcelo.reis@butantan.gov.br (M.d.S.R.); jose.patane@butantan.gov.br (J.S.L.P.); julia.cunha@butantan.gov.br (J.P.C.d.C.); 3 Laboratório de Toxinologia Aplicada, Instituto Butantan, São Paulo 05503-900, Brazil; 4 Departamento de Micro, Imuno e Parasitologia, Universidade Federal de São Paulo, São Paulo 04023-062, Brazil; marjorie.marini@gmail.com (M.M.); jose.franco@unifesp.br (J.F.d.S.); 5 Biomedicina, Centro Universitário São Camilo, São Paulo 04263-200, Brazil; 6 Laboratório de Ciclo Celular, Instituto Butantan, São Paulo 05503-900, Brazil

**Keywords:** *Trypanosoma cruzi*, genome, repeats, 3′UTR, multigenic family

## Abstract

*Trypanosoma cruzi* is the etiological agent of Chagas disease, which affects millions of people in Latin America. No transcriptional control of gene expression has been demonstrated in this organism, and 50% of its genome consists of repetitive elements and members of multigenic families. In this study, we applied a novel bioinformatics approach to predict new repetitive elements in the genome sequence of *T. cruzi*. A new repetitive sequence measuring 241 nt was identified and found to be interspersed along the genome sequence from strains of different DTUs. This new repeat was mostly on intergenic regions, and upstream and downstream regions of the 241 nt repeat were enriched in surface protein genes. RNAseq analysis revealed that the repeat was part of processed mRNAs and was predominantly found in the 3′ untranslated regions (UTRs) of genes of multigenic families encoding surface proteins. Moreover, we detected a correlation between the presence of the repeat in the 3′UTR of multigenic family genes and the level of differential expression of these genes when comparing epimastigote and trypomastigote transcriptomes. These data suggest that this sequence plays a role in the posttranscriptional regulation of the expression of multigenic families.

## 1. Introduction

The protozoan *T. cruzi* is the causative agent of Chagas disease and affects approximately 7 million people, mostly in Central and South America, where another 18 million people live at risk of infection [1]. This parasite exhibits a complex life cycle varying between the nonreplicative/infective form, known as the trypomastigote (bloodstream in mammalian host and metacyclic inside the vector), and the replicative forms, known as the amastigote (in the mammalian host) and epimastigote (in the invertebrate vector). Morphological and metabolic changes are observed among these life forms with the presence of distinct proteomic [2,3] and transcriptomic [4] profiles. However, the control of gene expression in *T. cruzi* relies largely on posttranscriptional and translation levels since transcription does not occur from a specific RNA pol II promoter for each gene but, rather, nonrelated genes are transcribed as a unique polycistron and then trans-spliced into individual mature mRNA molecules [5]. Therefore, other levels of gene expression regulation stand out, such as mRNA processing [6], translational repression [7,8,9], polysome recruitment [10], and codon adaptation [11,12]. In this scenario, noncoding DNA may also be involved as a regulatory element in mRNA expression [10,11,12,13].

Among coding and noncoding DNA, the *T. cruzi* genome presents at least 50% repetitive sequences, which include multigenic families, retrotransposons and subtelomeric repeats [14,15,16]. Of the repetitive DNA elements found within intergenic regions, most have no identified function to date. For example, satellite DNA is a 195 bp repetitive element that can be used as a *T. cruzi* infection marker in molecular diagnostics [17]; however, no function has been attributed to this sequence. Conversely, multigenic families mostly encode surface proteins involved in cell invasion as well as immune system evasion by *T. cruzi* [18]. The expression levels of these genes vary along the *T. cruzi* life cycle according to their function, but little is known about how they are regulated.

The 3′UTR (untranslated region) from mRNA and RNA binding proteins (RNA-BP) has emerged as a key factor in mRNA stability and protein expression level regulation in *T. cruzi*, including some proteins from multigenic families [19,20,21]. In this study, we developed a new strategy to identify new repetitive elements in the *T. cruzi* genome and found an intergenic repetitive sequence located downstream of many genes of multigenic families, such as mucin-associated proteins (MASPs) and trans-sialidases. Using RNAseq analysis, we confirmed that this sequence is present on the 3′UTRs of these mRNAs and is correlated with gene expression regulation, indicating that this repetitive sequence may have a cis-regulatory function on the expression of multigenic family mRNAs.

## 2. Materials and Methods

### 2.1. Filtering Steps for 150 bp Fragments

All of the 150 nucleotide sequences, obtained by a sliding window all over the genome sequence, were filtered through 4 different parameters: (1) Any sequence with at least one undefined nucleotide (N) was excluded; (2) Sequences with less than 10 copies were excluded; (3) Fragments with a significant match against the repetitive elements using RepeatMasker software (version 4.0.7) [22] were excluded (parameters “-species trypanosoma -pa 60 -u -xm -engine ncbi -excln”); and (4) Any repeat from multigenic family genes was excluded. Fragments were submitted to Blast-n alignment against an in house multigenic family database composed of 4999 genes (surface protease (GP63), mucin-associated surface protein (MASP), retrotransposons and trans-sialidase) from CL Brener (-S and -P haplotypes) using the parameters “-e-value 1e-72 -dust no -qcov_hsp_perc 100”.

### 2.2. Search Terms in the TriTryp Database

To establish the number of specific genes on *T. cruzi* strains Dm28c, Y, TCC, CL Brener haplotypes Esmeraldo-like (S), and non-Esmeraldo like (P), the following terms were searched in TriTrypDB [23]: “trans-sialidase”, “mucin-associated surface protein”, “TcMUC”, “mucin like”, “surface protease GP63”, “hypothetical protein”, “90 kDa surface protein”, “serine-alanine and proline-rich protein”, “dispersed gene family protein 1”, elongation factor 1-γ” and “retrotransposon hot spot protein”.

The searched terms “TcMUC” and “mucin like” were considered to be one category, “mucin”. Additionally, the terms “90 kDa surface protein” and “serine-alanine and proline-rich protein” were categorized under “90 kDa surface protein”.

### 2.3. Statistical Analysis

Student’s t-test was used for comparisons between samples using GraphPad Prism software (GraphPad Software Inc., San Diego, CA, USA).

### 2.4. Genomes Analyzed

Genome sequences from *T. cruzi* strains were downloaded from TritrypDB [23]: Dm28c 2018 (version 2018-05-30 release 46); Y C6 (version 2019-08-26 release 46); TCC (version 2018-05-30 release 46); CL Brener_S (version 2015-12-07 release 46); CL Brener_P (version 2015-12-07 release 46); marinkellei (version 1.0); Brazil A4 (version 2019-08-26 version 46); and Sylvio X10/1 (version 2017-03-18).

### 2.5. Monte Carlo Test of a 241 nt Repeat

To test whether the frequency of repeats associated with trans-sialidases was higher than expected by chance, we conducted a Monte-Carlo test [24], in which the total number of repeats found in the CL Brener S genome sequence (334) and in Dm28c strain (1117) were randomly re-inserted in the genome sequence. For each replicate, first a “fake” long and single chromosome was generated by concatenating the 41 chromosomes end-to-end from CL Brener_S and all 636 contigs of DM28c. Then, the repeats were randomly re-inserted between the first and last nucleotide of this “fake” chromosome. Finally, we determined how many of these re-inserted repeats had a trans-sialidase in their surroundings (either with the repeat falling into it or at 5′ and/or 3′). The rationale is that if the original number of repeats were to be inserted from scratch along chromosomes/contigs (for instance, by a natural biological process), then the distribution among the pseudoreplicates (334 draws per peudoreplicate for CL Brener S and 1117 for Dm28c) can be considered a reasonable indicator of the variability of the probability of random insertions near trans-sialidases. If the original value of repeats associated with such genes falls within the 95% highest density interval (HDI) of that distribution of the pseudoreplicates, then the hypothesis of random association with trans-sialidases in the original chromosomes/contigs cannot be discarded; on the contrary, if the original value of trans-sialidase associations is outside the 95% HDI (either higher or lower), then the hypothesis of random association with trans-sialidases can be ruled out at an α = 0.05 level.

### 2.6. RNA-seq Analysis

Coverage analyses were performed to investigate the relationships between the identified 241 nt repeat regions in the *T. cruzi* strains as well as their closest upstream and downstream genes and respective range regions. The expression profile for the repeat region, upstream and downstream genes and the region between them were quantified based on the RNA-Seq data from *T. cruzi* strains (CL_Brener Esmeraldo-Like, CL_Brener Non-Esmeraldo-Like, and YC6) from NCBI, for the two different stages of the parasite (epimastigote and trypomastigote) with two biological replicates for *T. cruzi* CL_Brener and three biological replicates for the other ones.

The accession numbers of SRAs from the NCBI of *T. cruzi* strains are as follows: CL_Brener epimastigote (SRX1643253, SRX1643239) and trypomastigote (SRX1643235, SRX1643234); Y epimastigote (SRX574896, SRX574895, SRX574894) and trypomastigote (SRX574893, SRX574892, SRX574891, SRX574890).

To quantify the expression profile of these regions, we aligned the samples to the reference genome using Hisat2 version 2.1.0 [25] with “−k1” parameter, which allows only one alignment per read. Then, the raw counts were quantified based on the alignment of reads to each genome strain with the tool multiBamCov, and the coverage of the region was estimated with coverageBed from bedtools version 2.26.0 [26]. The EdgeR version 3.28.1 [27] was used to estimate the average expression profile between the replicates calculated and the log2 fold change between the stages trypomastigote and epimastigote for every strain and for every region.

### 2.7. TPM Statistical Test

To test for a possible association of the repeat with the closest gene upstream in transcripts, in opposition to the closest gene downstream, we used previously available RNA-seq data from [databank]. Reads from three different lineages were used (CL Brener-P, CL Brener-S, and Y) for both epi- and trypomastigote forms, with transcripts per million (TPM) values averaged between forms within the same genome for each set of genic regions containing 5′ gene/repeat/3′ gene. We used the smallest difference between the TPM of the repeat to either the upstream or downstream gene’s TPM as a sign of the most likely posttranscriptional genic association of the repeat (“−1” if the smallest TPM difference was upstream, “+1” if downstream). The one-tailed sign test was then employed in R [28] to test the hypothesis of a stronger association to the upstream gene. This procedure was carried in two subsets of genes: (1) the set of four gene families to which repeats were found to be more associated with (trans-sialidases, etc.) appearing at either of the neighboring positions and (2) the remaining genic regions (“background regions”) in which none of the flanking genes was a member of those four gene families. Cases in which there was a second repeat flanking at the 5′ end of the upstream gene or at the 3′ end of the downstream gene were discarded.

## 3. Results

### 3.1. Identification and Distribution of a Novel DNA Repeat on the *T. cruzi* Genome

**Figure 1:**
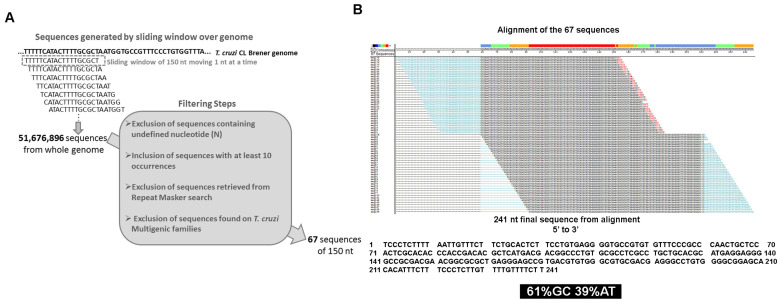
Strategy to identify a novel repetitive element in the *T. cruzi* CL Brener genome. (**A**) Schematic representation of the 150 nucleotide (nt) sliding window used to generate sequences covering all of the CL Brener genome and filtering steps used to exclude known repetitive elements. (**B**) Alignment of the 67 sequences of 150 bp obtained after the filtering steps that resulted in the consensus sequence of 241 nucleotides of the repetitive element.

The *T. cruzi* genome is composed of diverse repetitive elements that vary in size and copy number among different strains. The smallest high copy number element found in the *T. cruzi* genome is satellite DNA, which is 195 bp long with approximately 20,000 copies [29]. Therefore, we established a 150-nucleotide length to screen for new repetitive elements. We decided to start investigating both haplotypes (Esmeraldo like-S and non-Esmeraldo like-P) of the clone CL Brener genome sequence. This strain was used for the *T. cruzi* genome sequence project, and therefore, a considerable amount of information is available allowing future co-relation analysis. Moreover, the CL Brener strain has a hybrid origin containing haplotypes from different DTUs which could increase the robustness of our observations. Therefore, once the parental strains are from DTUs II and III, any DNA element found in both haplotypes would be more likely to be found in the genome sequences of other DTUs. In our approach, we used a sliding 150-nucleotide window along all chromosome sequences in each haplotype, moving it one nucleotide at a time, resulting in millions of 150-nucleotide fragments covering the entire genome of *T. cruzi* CL Brener (Figure 1A). A list with 52 million fragments was obtained and summarized, showing the frequency of 100% identical fragments that appeared during the window screening.

Furthermore, four sequential filtering steps were used to clean these data and isolate potential repetitive sequences. The first two steps excluded any fragment with at least one undefined (N) nucleotide, and then only the ones that appeared at least ten times on the list were selected. Next, the two additional filtering steps excluded the fragments with a significant match against the repetitive elements using RepeatMasker software and excluded any fragment from multigenic family genes. Therefore, the final list of 67 unique 150-nucleotide fragments was obtained (Figure 1A). Once all of the fragments were obtained by a sliding window, where sequential fragments were only one nucleotide apart, the final 67 fragments were aligned to determine whether they were independent repetitive sequences and/or part of a longer sequence. As shown in Figure 1B, all 67 sequences present 100% identity and aligned together, resulting in a consensus sequence composed of 241 nucleotides (Supplementary File S1). Therefore, using the 150-nucleotide sliding window and filtering and alignment steps, we identified a novel repetitive sequence on the *T. cruzi* genome that has not been described to date.

### 3.2. The 241 nt Repeat Is Enriched at Intergenic Regions of T. cruzi Genome Sequences

Since the 241 nt repeat was present in both CL Brener haplotypes, we next wanted to determine whether this repeat (i) is present in others *T. cruzi* strains and (ii) is present in other trypanosomatids. Blast-n search revealed that the 241 nt sequence is present in all searched *T. cruzi* strains, including the bat strain *T. cruzi marinkellei*, but it is absent in *Leishmania* and *Trypanosoma brucei* (Supplementary File S2).

To characterize this new repetitive element found in *T. cruzi*, we first checked the genome sequences of *T. cruzi* strains from different DTUs available in TritrypDB that were sequenced using long reads (PacBio technology) that provide more reliable assembly of these genomes (Table 1). Then, we chose one strain of each DTU to be analyzed, named as follows: Dm28c (TcI), Y (TcII), and TCC (TcVI). There are no strains from TcIII and TcIV sequenced by PacBio (Pacific Bioscience of California, Inc. Menlo Park, CA, USA) technology, and the TcV strain genome (Bug 2148) lacks annotation (Table 1). Even though the *T. cruzi marinkellei* genome was not sequenced by PacBio, we decided to perform some analyses on this strain in order to gain evolutionary insights into this repeat.

To identify the locations of this repetitive sequence on the genome, Blast-n searches on the *T. cruzi* genome sequences were performed. From the retrieved regions, only those with a minimum length of 140 bp and 95% identity to the 241 nt repeat were selected. The great majority of this repetitive element was found distributed on intergenic regions of the analyzed strains (Supplementary File S3): 100% in Dm28c (1117 of 1117), 99.5% in Y (742 of 746), 100% in TCC (1171 of 1171), 96.7% on CL Brener S (322 of 334), and 97.5% on CL Brener P (398 of 408). The repeats found in the genic regions (Supplementary File S4) were in genes of a hypothetical protein (four genes on Y, eight genes on CL Brener_S, and eight genes on CL Brener_P), ATPase (1 on CL Brener_S and 2 on CL Brener_P) and trans-sialidase (two on CL Brener_S). In fact, the identity between these two trans-sialidase sequences and the 241 nucleotide consensus sequence is the cause of the alignment break observed in Figure 1B (nucleotides 30–60 and 180–210), where the 150 nt fragments 100% identical to these trans-sialidases were eliminated after the multigenic family filtering step. Then, we further investigated the repeats located on intergenic regions.

**Figure 2:**
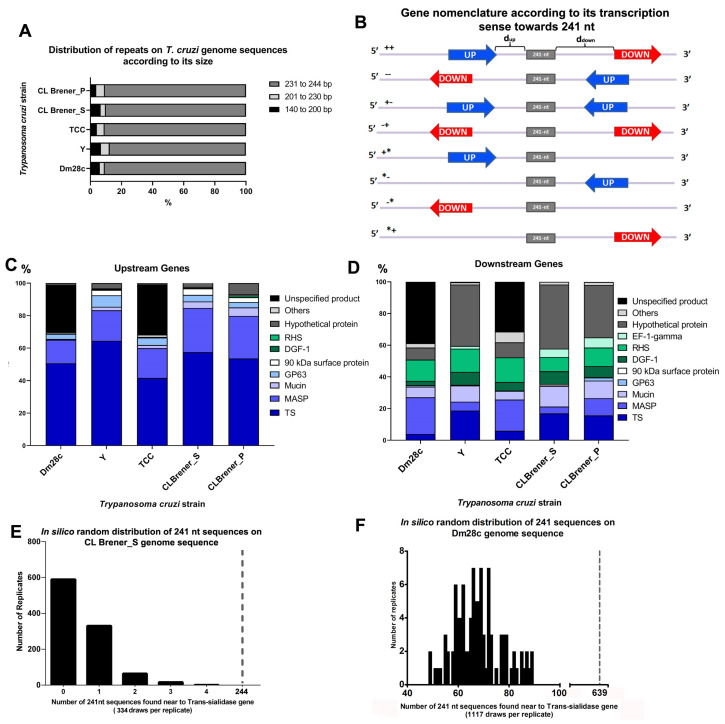
Localization of the 241 nt repetitive element in the *T. cruzi* CL Brener genome. (**A**) Consensus sequence of the 241 nt repeat that was submitted to Blast-n against the CL Brener genome, and the retrieved sequences with at least 95% identity and sizes from 140–244 nucleotides were selected. The graphic shows the frequency distribution of the retrieved sequences by their size in the genome sequence of *T. cruzi* strains Dm28c (TcI), Y (TcII), TCC (TcVI), and CL Brener S and P (TcVI). (**B**) Schematic representation of genes surrounding the 241 nt found inside the intergenic region. Genes were classified as upstream (UP) or downstream (DOWN) of the 241 nt repeat according to their transcription orientation. Distinct patterns of upstream and downstream genes in relation to the repeat are observed and indicated by the symbols ++, −−, +−, −+. In some cases, only one gene is associated with the repeat and is indicated by *+, +*, *−, and −*. Letter “d” indicates the distance between the repeat and upstream (d_up_)/downstream (d_down_) genes. (**C**) Percentage of genes upstream and (**D**) downstream of the 241 nt repeat on the *T. cruzi* genome sequences of Dm28c, Y, TCC, and CL Brener strains. (**E**) A total of 334 sequences of 241 nt were randomly distributed in the CL Brener_S genome sequence. The graph shows the number of repeats found near a trans-sialidase gene. The dashed line represents the number of repeats identified close to a trans-sialidase gene in the CL Brener genome (S haplotype). (**F**) A total of 1117 sequences of 241 nt were randomly distributed in the Dm28c genome sequence. The graph shows the number of repeats found near a trans-sialidase gene. The dashed line represents the number of repeats identified close to a trans-sialidase gene in the Dm28c genome Abbreviations: TS-trans-sialidase, MASP-mucin-associated surface protein, GP-glycoprotein, DGF-1-dispersed gene family-1, RHS-retrotransposon hot spot and EF-1 γ-elongation factor-1 γ.

The selected sequences from Blast-n were at least 140 nucleotides long, but approximately 90% of repetitive sequences found on the genome ranged from 231 to 244 nucleotides in DM28c, TCC and CL Brener and 87.5% in the Y strain (Figure 2A). Since the consensus sequence of this new repetitive element is 241 nucleotides long, we call these repeats a 241 nt repeat. These repeats located on intergenic regions were found interspersed throughout the genome sequences rather than organized in tandem in a head-to-tail fashion (Supplementary Files S5–S7), and they were present in most chromosomes from assembled genomes of CL Brener and Y strains (Table 2) (the DM28c and TCC genome sequences are not chromosome assembled). Even though larger chromosomes showed more copies of the repeat, its distribution was not proportional to chromosome size, as seen on Y strain chromosomes 2 and 3 (Table 2), where 3 copies are found on chromosome 2 and 73 copies are found on chromosome 3. Different copy numbers were also observed between CL Brener haplotypes, as seen on chromosome 40 (Table 2), where 34 repeats were found on the S haplotype and 8 were found on the P. In addition, the repeat distribution showed different profiles among the chromosomes, as it was observed at some chromosome edges in some chromosomes and concentrated in the middle in others (Supplementary Files S5–S7). Therefore, there was no preferential location along all chromosomes from the CL Brener and Y strains.

### 3.3. Regions Upstream and Downstream of the 241 nt Repeats Are Enriched in Surface Protein Genes

The interspersed distribution pattern of the 241 nt repeat and its intergenic location led us to investigate possible signs of correlation of this DNA repetitive element to nearby genes. First, these genes were classified according to their transcription orientation, where genes whose transcription orientation moved in the direction of the repeat were termed “upstream genes” and those whose transcription direction moved away from the repeat were termed “downstream genes” (Figure 2B), regardless of the strain being considered. From this analysis, different patterns could be observed, as shown in Figure 2B. Most 241 nt repeats were located between genes on the same polycistronic transcription unit (PTU) on the sense strand (indicated by ++) and anti-sense strand (indicated by −−), as shown on Table 3. In both cases (++ and −−), there were two genes, one gene upstream and the other downstream, flanking the 241 nt sequence. Fewer 241 nt repeats were located between convergent PTUs (indicated by +− in Table 3), and in this case, both adjacent genes were considered upstream. The 241 nt repeats were also located between divergent PTUs (indicated by −+ in Table 3), when both adjacent genes were denominated downstream. Additionally, some 241 nt copies had only one gene adjacent to it (indicated by +* and *− in Table 3), and these genes were always upstream genes. No 241 nt repeat was found with a single downstream gene close to it in the CL Brener strain (indicated by −* and *+ in Table 3); however, a few copies of this pattern were found on Dm28c, Y and TCC strains (Table 3).

The next step was to identify which genes surround the 241 nt repeat. Among upstream genes, the large majority were from multigenic families (trans-sialidase, MASP, mucin and GP63), that is, representing 96.6% of the genes in CL Brener_S, 91.2% in CL Brener_P, 95.6% in the Y strain, and 68.8% and 66.4% in the Dm28c and TCC strains, respectively. The remaining genes were mostly hypothetical proteins (representing 1.1% in Dm28c, 3.3% in Y, 0.8% in TCC, 2.5% and 6.9% in CL Brener S and P haplotypes, respectively) and some other genes (listed in Supplementary File S3) that collectively represent 1% in Dm28c, 0.9% in Y, 1.9% in TCC, and 0.8% and 1.7% in CL Brener S and P haplotypes, respectively (Figure 2C). When analyzing the percentage of these genes on the genome, these genes of multigenic families collectively represent 18.07% of the genes in Dm28c, 32.19% in Y, 16.15% in TCC, and 15.19% and 14.52% in CL Brener S and P, respectively. Additionally, the hypothetical protein genes from the genome correspond to 37.33% of the genes in Dm28c, 58.24% in Y, 37.14% in TCC, and 38.44% and 38.43% in CL Brener S and P, respectively, but only a small percentage was found upstream of the 241 nt repeat. Taken together, these data suggest that the 241 nt repeat is preferentially located near multigenic families along the genome and is not randomly distributed.

Once it was determined that the upstream genes are mostly composed of multigenic family genes and trans-sialidase (TS) genes are the most abundant among them, we tested whether the association between the 241 nt repeat and TS had biological relevance or was just a consequence of the random distribution of the 241 nt repeat in the genome. To address this question, we conducted a Monte Carlo test on genome sequences of CL Brener S (TcVI) and DM28c (TcI) strains that are highly divergent [30]. This test consists of random re-insertions of 241 nt repeats in the genome sequence according to the number of repeats originally identified in genome sequences (334 for CL Brener S and 1117 for Dm28c). In each replicate, random re-insertion was performed, and the number of trans-sialidase genes found flanking this repeat was counted. Monte Carlos analysis of CL Brener S showed that up to four TS genes were located close to the repeat after its random reinsertion into the CL Brener S genome (Figure 2E). As indicated by the dashed line in Figure 2E, the total number of TS genes found close to the 241 nt repeat in the *T. cruzi* CL Brener_S was 244, which is significantly higher than expected for the random distribution of the repeat (*p* < 0.01). For the Sylvio X10/1 strain, the Monte Carlo analysis showed that up to 89 repeats were found close to TS genes after the random re-insertion of the repeat, as shown in Figure 2F. Again, the number of TS genes flanking the 241 nt repeat (dashed line in Figure 2F) in the genome sequence of Sylvio X10/1 was significantly higher (*p* < 0.01) than that expected by chance distribution of the repeat in the genome. Therefore, these findings indicate that the proximity of repeats and the TS genes was not randomly distributed in the genomes analyzed and may have biological function.

We then analyzed the pattern of gene distribution in downstream genes, which was found to differ from that in upstream genes. The same multigenic family enriched upstream of the repeat (trans-sialidase, MASP, mucin and GP63) represented 35.5% of the downstream genes in the CL Brener_S, 39.8% in the CL Brener_P, 34.9% in the Y strain and 34.7% and 31.3% in the Dm28c and TCC strains, respectively. Additionally, two other multigenic family genes were among the downstream genes: DGF-1 (2.7% in Dm28c, 8.2% in Y, 5.4% in TCC, 8% and 7% in CL Brener S and P, respectively) and RHS (13.5% in Dm28c, 14.7% in Y, 15.6% in TCC, 9% and 11.7% in CL Brener S and P, respectively). The remaining genes were mostly genes for hypothetical proteins (7.6% in DM28c, 38.8% in Y, 9.5% in TCC, 40.5% in CL Brener_S and 33.1% on CL Brener_P). The Dm28c and TCC strains also presented “unspecific product” genes that represented 38.8% of the downstream genes in the first strain and 31.5% in the latter (Figure 2D). The higher amount of hypothetical protein genes and the lower amount of multigenic family genes among the downstream genes corroborate the closer relation of the repeat to upstream genes than to downstream genes.

In addition to the strains analyzed above, we also verified the repertoire of genes located upstream and downstream to the 241 nt repeats in *T. cruzi* Brazil A4 and Sylvio X10/1 strains (PacBio sequenced) as well as in the ancestral *T. cruzi marinkellei* strain. The Brazil A4 strain and *T. cruzi marinkellei* presented similar repertoires in their upstream genes (Supplementary Files S8 and S9), where trans-sialidase genes represented the great majority (73.7% in Brazil A4 and 75% in *T. cruzi marinkellei*), followed by MASP genes (13% in Brazil A4 and 6.3% in *T. cruzi marinkellei*). Other multigenic family genes were also observed among the upstream genes (Supplementary Files S8 and S9) and, collectively, the multigenic family genes (TS, MASP, mucin and GP63) represented 93.7% and 85% of the upstream genes from the Brazil A4 strain and *T. cruzi marinkellei*, respectively. When analyzing genes found downstream to the repeat, the repertoires found in Brazil A4 and *T. cruzi marinkellei* were similar to those found in previously analyzed strains but differed in the amount of multigenic family genes (Supplementary Files S8 and S9). In the ancestral *T. cruzi marinkellei*, MASP genes comprised 36.8%, followed by trans-sialidase and hypothetical protein genes (both representing 13.2% of the downstream genes). In the Brazil A4 strain, the most abundant genes among downstream genes were hypothetical protein genes (35.8%) and trans-sialidase (24.1%) (Supplementary Files S8 and S9). Surprisingly, *T. cruzi* Sylvio X10/1 strain analysis revealed different genes flanking the 241 nt repeat (Supplementary Files S8 and S9). Bacterial neuraminidase repeat (BNR)-like domain genes were the most abundant genes among the upstream genes (49.82%) and the second most abundant among the downstream genes (27.86%). The concanavalin A-like lectin/glucanases superfamily represented 20% of the upstream genes and 14.5% of the downstream genes, while leishmanolysin represented 7.64% of the upstream genes and 8.4% of the downstream genes. RHS (5.09% and 3.44%, of the upstream and downstream genes respectively), EF1-γ (4.73% and 2.29% of the upstream and downstream genes respectively), trans-sialidase (1.09% and 0.38% of the upstream and downstream genes respectively) and DGF-1 (4.73% of the upstream genes) also flanked the repeat. Genes identified as “unspecific products” comprised 35% of the downstream genes (Supplementary Files S8 and S9). The fact that the 241 nt repeat is exclusive to *T. cruzi* and that the bat subspecies *T. cruzi marinkellei* presented similar composition illustrates how ancient this repeat found among *T. cruzi* is and reinforces its potential biological role.

### 3.4. The 241 nt Repeat Is Found Closer to Upstream Genes and May be Part of the 3′UTR of Trans-Sialidase Gene mRNA

**Figure 3:**
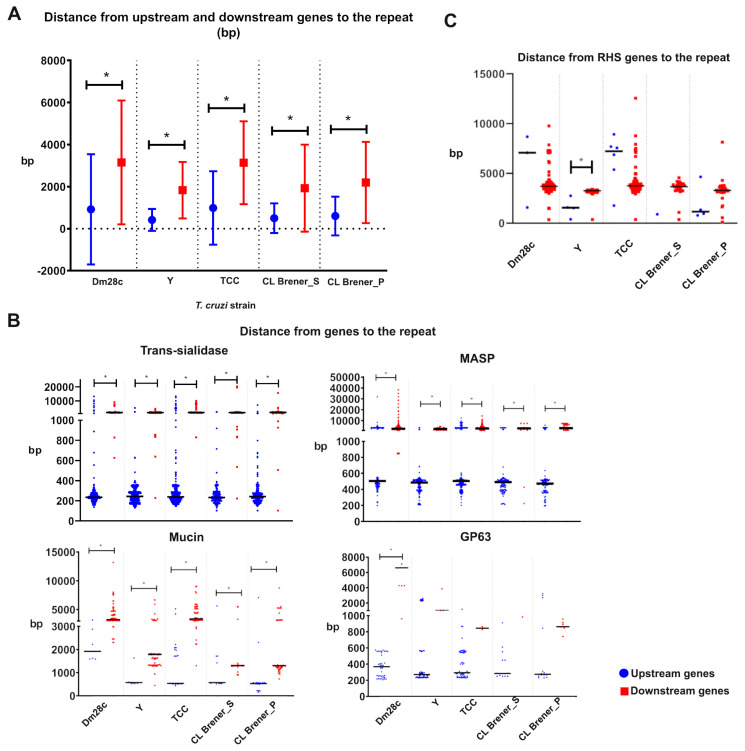
Distance from 241 nt repeats of upstream and downstream genes. The distances of each gene upstream (blue symbols) and downstream (red symbols) of the 241 nt repeat are plotted on the graph. Horizontal bars indicate the mean, and * indicates a *p* value < 0.001 from the Student’s t-test. (**A**) Distance from the 241 nt repeat to upstream genes and downstream genes on Dm28c, Y, TCC and CL Brener Esmeraldo-Like haplotype and non-Esmeraldo-Like haplotype. (**B**) Distance from 241 nt to the four main multigenic families of genes among upstream and downstream genes on Dm28c, Y, TCC and CL Brener genome sequences. (**C**) Distance from the repeat to hypothetical proteins.

The intergenic location of the 241 nt repeat and the different gene profiles of upstream and downstream genes of the repeat motivated us to determine the distance between the 241 nt and the upstream and downstream genes (indicated by “d_up_” and “d_down_” on Figure 2B). Comparing the distances from the 241 nt repeat to the upstream and downstream genes, it was observed that the 241 nt repeat was found to be significantly closer to upstream genes than to downstream genes in all of the *T. cruzi* strains analyzed (Figure 3A and Supplementary Files S8 and S9) including *T. cruzi* marinkellei (Supplementary Files S8 and S9), with the exception of Sylvio X10/1 strain (Supplementary Files S8 and S9).

When the distance between the repeat and each multigenic family was analyzed, the 241 nt repeat was found to be significantly closer to some upstream multigenic families, including trans-sialidase, MASP and mucin, on all strains analyzed. The GP63 from upstream genes was closer to the repeat than from downstream genes; however, it was significant only in the Dm28c strain (Figure 3B). In contrast, the distance from RHS to the repeat was different among strains, while in Y and CL Brener P strains, the repeat was closer to upstream RHS genes (significant only in the Y strain), and in Dm28c and TCC, the repeat is closer to RHS from downstream genes (Figure 3C).

**Figure 4:**
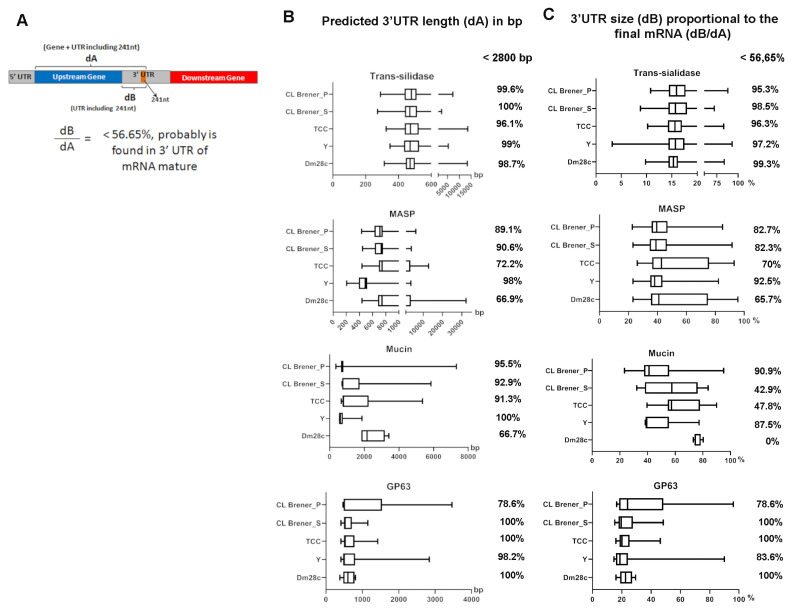
The 241 nt repeat is found in the 3′UTR of upstream genes. (**A**) Schematic representation of upstream and downstream genes to the 241 nt sequence and distances used to predict the 3′UTR length (dA). (**B**) Predicted 3′UTR including the 241 nt sequence (distance dA is represented on “b”). The percentage of genes that predicted 3′UTR represents less than 2800 bp of the mRNA and is listed on the left of the graphs. (**C**) The predicted 3′UTR size (distance dB is represented on “b”, which includes the 241 nt sequence) in proportion to the final full mRNA was plotted. The percentage of genes that predicted 3′UTR represents less than 56.65% of the mRNA is listed on the left of the graphs. In (**B**) and (**C**), the four major representative genes among upstream and downstream genes are represented. Abbreviations: MASP-mucin-associated surface protein and GP-glycoprotein.

The proximity of the 241 nt repeat to upstream genes raised the question whether this repeat could be transcribed as part of the 3′UTRs mRNA of upstream genes. Since the UTR length of mRNA from *T. cruzi* varies in size, ranging from 17 to 2800 nucleotides, and is generally limited to 56.65% of the final mRNA [31], we used these two pieces of information to infer the possible presence of the 241 nt repeat in the 3′UTR of trans-sialidase, MASP, mucin and GP63 final mRNA. To this end, we first calculated the distance from the first nucleotide after the stop codon (of upstream gene) to the last nucleotide of the 241 nt sequence, as shown in Figure 4A (dB), and then analyzed the proportion of genes where dB was lower than 2800 bp. Second, we calculated the distance from the first nucleotide of upstream genes to the last nucleotide of the 241 nt repeat (dA in Figure 4A) and calculated the ratio of dB/dA. Then, the proportion of genes where dB/dA was lower than 56.65% was investigated. Therefore, when dB is lower than 2800 bp and the dB/dA ratio is lower than 56.65%, it is possible that the repeat is enclosed into the 3′UTR of the final mRNA.

Over 96% of the trans-sialidase genes from DM28c, Y, TCC, and CL Brener showed a dB lower than 2800 bp and a dB/dA ratio lower than 56.65% (Figure 4B,C). For MASP genes, there was variation among the strains: In Dm28c, 66.9% of the MASP genes showed a dB lower than 2800 bp (Figure 4B), and 65.7% of the MASP genes showed a dB/dA ratio lower than 56.65% (Figure 4C). In Y strains, 98% of the MASP genes showed a dB lower than 2800 bp (Figure 4B), and 92.5% of the MASP genes showed a dB/dA lower than 56.65% (Figure 4C). In TCC, 72.2% of the MASP genes showed a dB lower than 2800 bp (Figure 4B), and 70% of the MASP genes showed a dB/dA lower than 56.65% (Figure 4C). In the CL Brener strain, 90.6% (S) and 89.1% (P) of the MASP genes showed a dA lower than 2800 bp (Figure 4B), and 82.3% (S) and 86.7% (P) of the MASP genes showed a dB/dA ratio lower than 56.65% (Figure 4C). The analysis of GP63 genes showed that 100% of the GP63 genes from Dm28c, TCC and CL Brener S had a dB lower than 2800 bp (Figure 4B) and a dB/dA lower than 55.65% (Figure 4C). In the Y strain, 98.2% of GP63 genes showed a dB lower than 2800 bp (Figure 4B), and 83.6% of the GP63 genes showed a dB/dA ratio lower than 56.65% (Figure 4C). In CL Brener P, 78.6% of the GP63 genes showed a dB lower than 2800 bp (Figure 4B) and a dB/dA ratio lower than 56.65% (Figure 4C).

As can be observed from the results described above, trans-sialidase, MASP and GP63 genes (from the four strains) had similar proportions of genes with a dB lower than 2800 bp and a dB/dA ratio lower than 56.65%. However, the analyzed mucin genes presented greater differences between the dB and dB/dA ratio analyses. In Dm28c, 66.7% of mucin genes showed a dB lower than 2800 bp (Figure 4B), and 0% of mucin genes showed a dB/dA lower than 56.65% (Figure 4C). In the Y strain, 92.5% of mucin genes showed a dB lower than 2800 bp (Figure 4B), and 83.6% of mucin genes showed a dB/dA lower than 56.65% (Figure 4C). In TCC, 91.3% of mucin genes showed a dB lower than 2800 bp (Figure 4B), and 47.8% of mucin genes showed a dB/dA ratio lower than 56.65% (Figure 4C). In CL Brener, 92.9% (S) and 95.5% (P) of mucin genes showed a dB lower than 2800 bp (Figure 4B), and 42.9% (S) and 90.9% (P) of mucin genes showed a dB/dA ratio lower than 56.65% (Figure 4C).

These data suggest that the 241 nt repeat can be part of the 3′UTR of the final mRNA of most trans-sialidase, MASP and GP63 genes. The mucin genes analyzed here showed a lower proportion where the 241 nt repeat is located in the 3′UTR. Additionally, mucin is the multigenic family with the fewest genes in the genome associated with the repeat (approximately 5%; Supplementary File S8); thus, any function of this repeat may have a minor role on mucin genes.

### 3.5. The 241 nt Repeats Are Found Significantly Expressed in Transcriptomes and Highly Correlated to the mRNA 3′UTR Sequence

To answer the question whether the 241 nt repeat is indeed expressed in the 3′ UTR of the final RNA and the possible role of this repeat in gene expression, transcriptome datasets available in GenBank [32] were analyzed. First, we selected all RNA-seqs of epimastigote and trypomastigote forms with at least two replicates each. Only the Dm28c, Y and CL Brener strains were available, and only the Y and CL Brener transcriptome data could be analyzed due to the percentage of aligned reads (Supplementary File S10). Therefore, RNAseq data of the *T. cruzi* CL Brener strain (Franco G.R., unpublished data) and Y strain [33,34] from epimastigote and trypomastigote forms were aligned against a reference genome sequence, coverage analysis was performed, and the expression profiles of the 241 nt repeat and surrounding regions were obtained.

**Figure 5:**
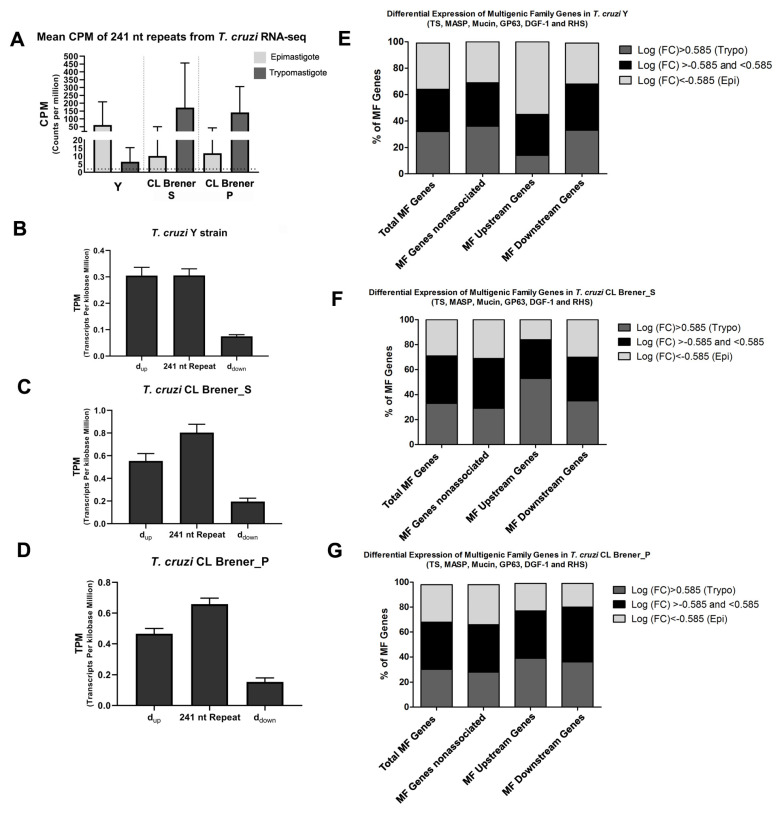
RNA-seq analysis from Y and CL Brener strains of *T. cruzi*. After RNA-seq alignment with reference genomes (Y and CL Brener strains), the coverage and expression profile were obtained from 241 nt repeats and surrounding regions. (**A**) CPM values from 241 nt repeats in epimastigotes (light gray) and trypomastigotes (dark gray) (CL Brener and Y strain). The dashed line indicates the cut-off (2). (**B**–**D**) TPM values from transcripts aligned in the 241 nt repeat, d_up_ and d_down_ segments of CL Brener_S (**B**), CL Brener_P (**C**) and Y strain (**D**). (**E**–**G**) Gene expression profile of multigenic families associated and nonassociated with the 241 nt repeat. The Log2(FC) values of the trypomastigote/epimastigote ratios were calculated, and a FC of 1.5 x was chosen as the cut-off. A Log2(FC) > 0.585 indicates genes upregulated in trypomastigotes (dark gray), a log2(FC) < −0.585 indicates genes upregulated in epimastigotes (light gray) and a log2(FC) between −0.585 and 0.585 indicates nondifferential expression (black). Abbreviations: CPM-counts per million; TPM-transcripts per million, FC-fold change, TS-trans-sialidase, MASP-mucin-associated surface protein, DGF-1-dispersed gene family, RHS-retrotransposon hot spot and MF-multigenic family.

To determine the presence of reads covering the 241 nt repeat sequence, the counts per million reads mapped (CPM) parameter was used. The cut-off value of 2 was established, and CPMs over 2 were considered as a significant expression of the analyzed region. Figure 5A shows the CPM values from epimastigote and trypomastigote forms of Y and CL Brener strains, and the great majority of the repeats are expressed in epimastigote forms (96.9% in Y and 70.6% and 76.5% in CL Brener S and P, respectively) and trypomastigote forms (66.4% in Y and 100% in CL Brener S and P). In fact, the CPM mean is over 60 in both strains (in the epimastigote form of the Y strain and in the trypomastigote form of the CL Brener strain). Thus, the 241 nt repeats are not just a repetitive element on the genome but also are expressed as constituent of the final RNA.

Once the presence of the 241 nt repeat is confirmed in mRNAs, we then analyzed if the 241 nt repeat was indeed in the 3′ UTR of multigenic family genes (trans-sialidase, MASP, mucin and GP63), as predicted by the genomic analysis. For that, the TPM of three regions were considered: the 241 nt repeat, the region between the repeat and upstream gene (indicated by d_up_ in Figure 2B) and the region between the repeat and downstream gene (indicated by d_down_ in Figure 2B). Additionally, only the 241 nt repeats flanked by one upstream gene and one downstream gene were considered (patterns ++ and −− of Figure 2B and Table 3).

Some background information is provided below:

(i) The TPM parameter interprets the transcriptional abundances of determined regions, allowing comparison of the proportion of reads among mapped regions because the TPM normalizes the depth and length of the sequencing data.
(ii) The d_up_ encloses the 3′ UTR, while the d_down_ corresponds to the entire region between the nucleotide just after the 241 nt repeat to the last nucleotide before downstream gene. Therefore, the d_down_ encloses the segment transcribed from the genome but lost after trans-splicing as well as the 5′ UTR of the downstream gene. Thus, d_up_ will have higher amounts of transcripts (higher TPM), and d_down_ will have lower amounts of transcripts (lower TPM).

Therefore, the TPM analysis rationale was that if the 241 nt repeat is part of the mRNA 3′ UTR, the TPM from the repeat and d_up_ would be more similar, but if the 241 nt repeat is not part of the 3′ UTR, the repeat TPM would be similar to the d_down_ TPM. Figure 5B–D show the mean TPM values from the Y strain (Figure 5B), CL Brener_S (Figure 5C) and CL Brener_P (Figure 5D). The TPMs of the 241 nt repeat regions are higher than the TPMs of d_down_, and repeat TPM values are closer to the d_up_ TPMs. To assess whether there is an association of the TPM value from repeat and d_up_, a statistical test was applied.

In the CL Brener (S and P) and Y strains, the 241 nt repeats were significantly associated with d_up_ according to the one-tailed sign test (*p*-value < 0.01 for both strains). There was a strong association between the repeat and the d_up_ corresponding to the genes of multigenic families (trans-sialidase, MASP, mucin and GP63). The remaining genes showed no significant association with the repeats in CL Brener-S (*p* = 0.82), borderline significance for CL Brener-P (*p* = 0.046), and a significant association for the Y genome (*p* = 0.002), although in all cases, the association was not as strong as that in the set of the four gene families (trans-sialidase, MASP, mucin and GP63).

Additionally, the regions corresponding to d_up_ and the 241 nt repeat were analyzed in terms of their coverage, and most of these regions on CL Brener and Y strains were completely covered (Supplementary File S11). Taken together, these data strongly indicate that the 241 nt repeat is indeed expressed in the 3′ UTR of the genes of multigenic families such as trans-sialidase, MASP, mucin and GP63.

### 3.6. Distinct Expression Profile Between the Epimastigote and Trypomastigote of Genes Is Associated with the 241 nt Repeat

The RNA-seq data also allowed us to quantify the expression profile of epimastigotes and trypomastigotes from CL Brener and Y strains. In that manner, six multigenic family (MF) genes were selected, and four were found to be enriched among upstream genes (trans-sialidase, MASP, mucin and GP63) plus DGF-1 and RHS (enriched among downstream genes). For this analysis, only upstream genes and downstream genes from patterns ++ and −− (Figure 2B) were considered, and all genes annotated as “pseudogene” were excluded.

The log2(fold change, FC) ratio of trypomastigote/epimastigote from the six multigenic family genes were calculated, and the results are summarized in Figure 5E–G. A FC (fold change) of 1.5 was established, and thus, genes were considered upregulated in trypomastigotes when the log2(FC) was higher than 0.585 and upregulated in epimastigotes when the log2(FC) was lower than −0.585. Figure 5E–G shows the percentage of genes differentially expressed in epimastigotes or trypomastigotes as well as the percentage of genes not differentially expressed. The MF genes were organized into four different groups so that the FC could be compared among them: 1. total MF genes from the genome; 2. MF genes that are not flanking the 241 nt repeat (nonassociated); 3. MF genes located upstream to the 241 nt repeat; and 4. MF genes located downstream to the 241 nt repeat. Analyzing the FCs of the genes of the six multigenic families of *T. cruzi* Y strain, a similar distribution among the three FC ranges was observed (Figure 5E): 32% of the total MF genes were upregulated in trypomastigotes, 35% were upregulated in epimastigotes, and 35% had no differential expression.

MF genes not associated with the 241 nt repeat and MF genes among downstream genes had similar results to the total MF genes; however, the group of MF genes among the upstream genes showed a decrease in the percentage of genes upregulated in trypomastigotes (~14%).

In *T. cruzi* CL Brener, the total MF genes showed that ~38% in S and ~39% in P of the genes are not upregulated in epimastigotes and trypomastigotes; meanwhile, ~29% in S and ~30% in P are upregulated in epimastigotes, and ~33% in S and ~31% in P are upregulated in trypomastigotes. Slight changes were observed among MF nonassociated genes; however, MF among upstream genes showed an increase in the percentage of genes upregulated in trypomastigotes (~53% in S and ~39% in P) together with a decrease in the percentage of upregulated genes in epimastigotes (~16% in S and ~22% in P). Additionally, MF among the downstream genes of CL Brener P showed a decrease in epimastigote upregulated genes (~20%), while in CL Brener S the percentage of MF among downstream genes was similar to that of the MF nonassociated genes.

These changes in differential expressed genes (DEG) of MF genes containing the 241 nt repeat in 3′ UTR strongly point to a relevant role of the 241 nt repeat in gene expression regulation among different life cycles of *T. cruzi*.

## 4. Discussion

The *T. cruzi* genome presents a highly repetitive DNA fraction that comprises at least 50% of its genome [14]. Apart from multigenic families, which encode mostly surface proteins and have some of their functions established, most of the repetitive elements in the *T. cruzi* genome do not yet have a defined function. Through a new approach of genome screening using a sliding window of 150 nucleotides, sequential filtering steps, and alignment of the resulting sequences, a resulting repetitive sequence of 241 nts was identified and mapped in each chromosome of clone CL Brener through Blast-n search in TriTrypDB. Further analysis showed that the 241 nt repeat is found in all strains of *T. cruzi* as well as in the ancestral strain *T. cruzi marinkellei*. This sequence was found to be distributed on almost all chromosomes of CL Brener and Y strains as an interspersed repetitive element and enriched in chromosomes with high concentrations of multigenic families, such as chromosomes 18, 28, 38, and 48 from CL Brener S [35]. However, the 241 nt repeat seems to not be randomly distributed along the genome, as it has a close relationship with its upstream genes (defined according to its transcription orientation). Analyzing the distance between the 241 nt and the upstream and downstream genes, a significantly shorter distance was observed from this element to upstream genes. Furthermore, the repertoire of genes found upstream from the 241 nt repeat was mostly composed of surface protein genes in all analyzed strains (Dm28c, Y, TCC, CL Brener, Brazil A4) and *T. cruzi marinkellei*. Surprisingly, in the Sylvio strain, we did not observe the proximity of the 241 nt repeat to upstream genes, and genes flanking the repeat were not the same as those found in all other strains. Since the other strains from TcI, TcII, and TcVI as well as the ancestral *T. cruzi marinkellei* presented the same repertoire of genes flanking the 241 nt repeat, the differences in the repertoire found in the Sylvio strain could be from genome assembly that is fragmented in repetitive regions [30].

In the *T. cruzi* genome, repeated elements (micro- and minisatellite repetitive DNA, retroelements) and multigenic families encoding surface proteins (TS, GP63, MASP, and mucins), DGF-1 and RHS are located in large nonsyntenic regions [16,36,37], also named disruptive compartments [37]. Since the 241 nt repeats are primarily associated with the surface protein genes RHS and DGF-1, they have been mapped on the nonsyntenic regions of the genome. Interestingly, even the hypothetical protein genes carrying the 241 nt repeat were mapped to this region. Taken together, these results suggest that the duplication of 241 nt repeats occurred together with the expansion of multigenic families in *T. cruzi*.

Transcriptome data analysis showed that the 241 nt repeat is indeed expressed in epimastigote and trypomastigote forms of *T. cruzi* (strains Y and CL Brener). Moreover, the 241 nt repeat is transcribed as part of 3′ UTR of trans-sialidases, MASP, mucin and GP63 and its presence seems to be involved in gene expression regulation. Gene expression analysis of trypomastigotes and epimastigotes (Y and CL Brener) indicated that MF genes associated with the 241 nt repeat are differentially expressed when compared to the MF genes nonassociated with the repeat. In CL Brener strain, a higher percentage of MF genes among upstream genes are upregulated in trypomastigotes, while in the Y strain, the MF genes among upstream genes are downregulated in trypomastigotes. However, the molecular bases that contribute to different expression patterns of genes harboring the 241 nt repeat in each of the two strains (Y and CL Brener) remain unknown.

Three findings reinforce the possibility of a biological function for this repeat: (i) the presence of the 241 nt repeat in the genomes of all analyzed DTUs and in the ancestral *T. cruzi marinkellei*; (ii) the conserved repertoire of genes flanking the 241 nt repeat in different strains and in the ancestral subspecies; and (iii) the presence of the 241 nt in the 3′ UTR region of MF genes whose expression changes in different forms of *T. cruzi* life cycle. Therefore, we propose that the 241 nt repeat could serve as a cis-regulatory element on mRNA, playing a role in the posttranscriptional regulation of surface proteins of *T. cruzi*. UTR segments are involved in gene expression regulation, regulating mRNA transcription to mRNA decay [38] and in the interaction of mRNA with other RNA molecules [39]. Diverse elements in the 3′ UTR region have been described to have cis-regulatory functions in gene expression [40,41] not only in later divergent eukaryotes but also in trypanosomatids [10,20,42,43,44]. In *T. cruzi*, for example, mRNAs harboring a 43-nt U-rich element in its 3′UTR are upregulated in amastigote forms. This U-rich sequence is subject to TcUBP1 (a RNA binding protein) binding, which leads to mRNA destabilization in epimastigotes and mRNA expression in amastigotes [45]. Moreover, in *Leishmania,* a 450-nt sequence was identified and showed the cis-regulatory function of mRNAs, causing an amastigote stage-specific expression of mRNA harboring it on its 3′ UTR [10]. Further experiments are necessary to investigate the proposed biological function for this repeat that, if confirmed, will contribute to the understanding of the controlled expression of genes in *T. cruzi*, a medically important organism that presents a unique system of gene expression among eukaryotes.

## 5. Conclusions

Through a new approach of genome screening that involves nucleotide window sliding, filtering steps, and sequence alignment, a novel repetitive sequence of 241 nts was identified. The 241 nt element (named 241 nt repeat) is not found on the *T. brucei* and *Leishmania sp* genomes, and it is interspersed on almost all chromosomes of *T. cruzi* (Y and CL Brener strain). The repertoire of genes found upstream from the 241 nt repeat was mostly composed of surface protein genes encoding trans-sialidases, MASP, mucins and GP63 protease. Since (i) this new repeat was found to be transcribed as part of the 3′ UTR of mRNAs of these multigenic families and (ii) MF harboring the 241 nt repeat presents a gene expression profile different from those not harboring the repeat, the involvement of the 241 nt repeat in the control of gene expression in *T. cruzi* is strongly suggested.

## Figures and Tables

**Table 1:** *T. cruzi* genomes available in TritrypDB. With the exception of *T. cruzi marinkellei* (sequenced by Illumina technology-Illumina Inc., San Diego, CA, USA) and the CL Brener strain (reference genome sequenced by whole genome shotgun assembly), the strains were sequenced by PacBio technology. NA: Not Annotated/* chosen strains for formal analysis.

DTU	B7	TcI			TcII	TcV	TcVI		
*T. cruzi* strain	Marinkellei	Dm28c *	Brazil A4	Sylvio X10/1	Y C6 *	Bug2148	TCC *	CL Brener S *	CL Brener P *
Chr count	0	0	43	47	40	0	0	41	41
Contig count	16783	636	359	0	226	929	1236	0	0
Genome size (Mbp)	38.65	53.27	45.56	41.38	47.22	55.16	87.06	32.53	32.53
Total gene count	10282	19112	18779	20684	17713	NA	29302	10596	11106

**Table 2:** Number of 241 nt repeats found on each chromosome of *T. cruzi* CL Brener.

	Y C6		CL Brener		
			S and P	S	P
Chr	Chr Size (kb)	N° of Repeats	Chr Size (kb)	N° of Repeats	N° of Repeats
1	2,950,016	146	77,958	0	0
2	1,943,341	3	151.74	2	1
3	1608.8	73	196,644	1	0
4	1,578,048	2	200,401	3	1
5	1,465,819	45	227,319	0	1
6	1,365,397	24	389,024	3	2
7	1238.82	2	391,095	9	3
8	1,238,493	2	393,493	1	2
9	1,233,391	8	509,634	0	1
10	1,196,034	6	518,846	0	0
11	1,179,968	2	526.14	4	1
12	1,154,569	3	533,093	2	4
13	1,073,329	10	558,364	1	0
14	1041.73	6	598,625	5	7
15	973,991	10	612,853	4	17
16	931,817	0	646,207	11	16
17	919,065	3	648,584	8	4
18	889,019	10	655,081	28	23
19	879,731	15	671,453	7	1
20	835,455	23	656,799	15	5
21	802.19	3	704,149	4	9
22	794,882	13	710,778	3	2
23	771,598	18	655,477	5	7
24	748,092	8	779,922	6	10
25	747,041	8	822,374	9	24
26	713.53	17	801,422	9	6
27	704,292	0	850,241	0	0
28	683,656	4	853,233	12	27
29	683,261	6	870,934	12	18
30	618,893	9	863,882	2	2
31	613,739	9	947,473	4	6
32	587,789	6	968,069	0	1
33	572,88	19	1,041,172	3	13
34	565,606	1	1,065,764	4	1
35	563,146	6	1,186,946	1	4
36	542,602	21	1,180,744	2	1
37	354,446	3	1,355,803	3	2
38	332,206	0	1,444,805	24	67
39	241,231	2	1,854,104	3	3
40	239,696	5	2,036,759	34	8
41			2,371,736	90	108
unplaced contig		201			\
Total		752		334	408

**Table 3:** Number of repeats found in the intergenic region of each *T. cruzi* strain. The + symbol indicates gene transcription from the sense strand, and the − symbol indicates gene transcription from the anti-sense strand. The left symbol represents the gene at the left side of the repeat, and the right symbol represents the gene at the right side of the repeat.

	TcI	TcII	TcVI		
	Dm28c	YC6	TCC	CLBrener_S	CLBrener_P
++	472	349	472	150	190
−−	512	351	516	148	163
+−	94	38	103	22	45
−+	2	1	11	2	3
+*	11	4	24	4	1
*−	18	6	30	8	6
−*	5	1	5	0	0
*+	3	2	10	0	0
Total	1117	752	1171	334	408

## Supplementary Materials

The following are available online at https://www.mdpi.com/2073-4425/11/10/1235/s1; Supplementary File S1. DNA sequence of the 241 nt repeat; Supplementary File S2. Number of retrieved sequences from the 241 nt Blast-n search on available genome sequences of *Leishmania*, *Trypanosoma brucei* and *T. cruzi* from TritrypDB; Supplementary File S3. Graphs representing the frequency and location of 241 nt repeats on each chromosome of the Esmeraldo-like haplotype from the *T. cruzi* CL Brener genome; Supplementary File S4. Graphs representing the frequency and location of 241 nt repeats on each chromosome of the non-Esmeraldo-like haplotype from the *T. cruzi* CL Brener genome; Supplementary File S5. Graphs representing the frequency and location of 241 nt repeats on each chromosome of the *T. cruzi* Y strain (YC6 from TritrypDB). Supplementary File S6. List of 241 nt repeats found in intergenic region of *T. cruzi* genome sequences from the following strains: Dm28c, Y, TCC and CL Brener; Supplementary File S7. List of 241 nt repeats found inside coding regions of *T. cruzi* genome sequences from the following strains: Dm28c, Y, TCC and CL Brener; Supplementary File S8. Tables containing (i) the total number of genes found upstream and downstream to the 241 nt repeat and their representation in percentage, (ii) List of genes and their percentages among the total genes from the genome sequence and (iii) genes from the genome found upstream and downstream to the 241 nt repeat (%); Supplementary File S9. Repertoire of genes flanking the 241 nt repeat in *T. cruzi* Brazil A4, Sylvio X10/1 and *T. cruzi* marinkellei and a graph showing the distance from the 241 nt repeat to upstream and downstream genes of *T. cruzi* Brazil A4, Sylvio X10/1 and *T. cruzi* marinkellei; Supplementary File S10. Table containing information of the RNAseq alignment of *T. cruzi* CL Brener, Y and Dm28c strains. Supplementary File S11. Table containing the percentage of d_up_ region covered by RNAseq reads of *T. cruzi* CL Brener and Y strains.
