# Supplementary figures and images for: Identification of Novel Interspersed DNA Repetitive Elements in the Trypanosoma cruzi Genome Associated with the 3′UTRs of Surface Multigenic Families
Source: Genes (Basel). 2020 Oct 21;11(10):1235. doi: 10.3390/genes11101235 (PMC7593948; doi:10.3390/genes11101235)

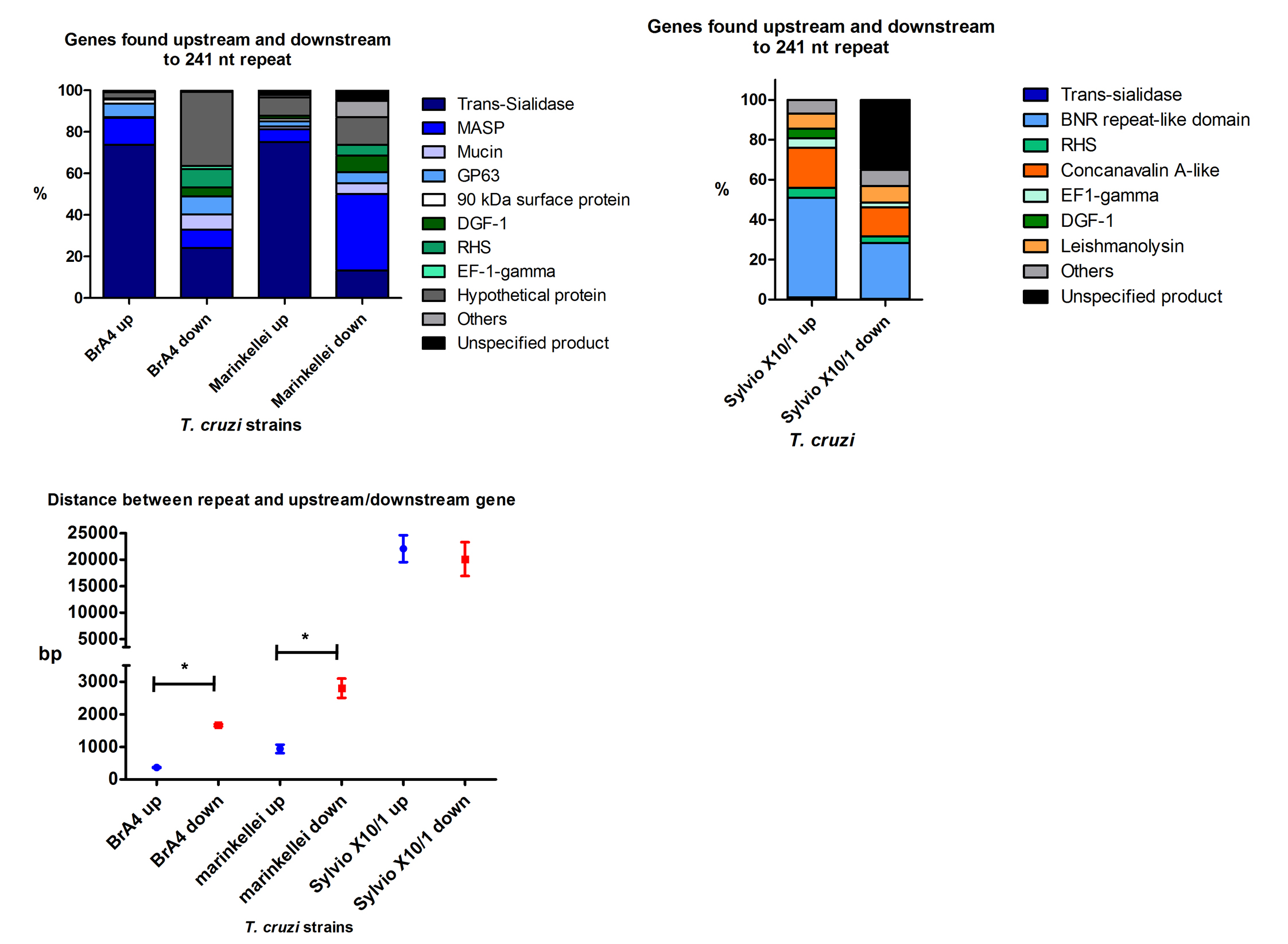

Supplement: Supplementary file 1 [file genes-11-01235-s001.zip › Supplementary File 9.jpg]

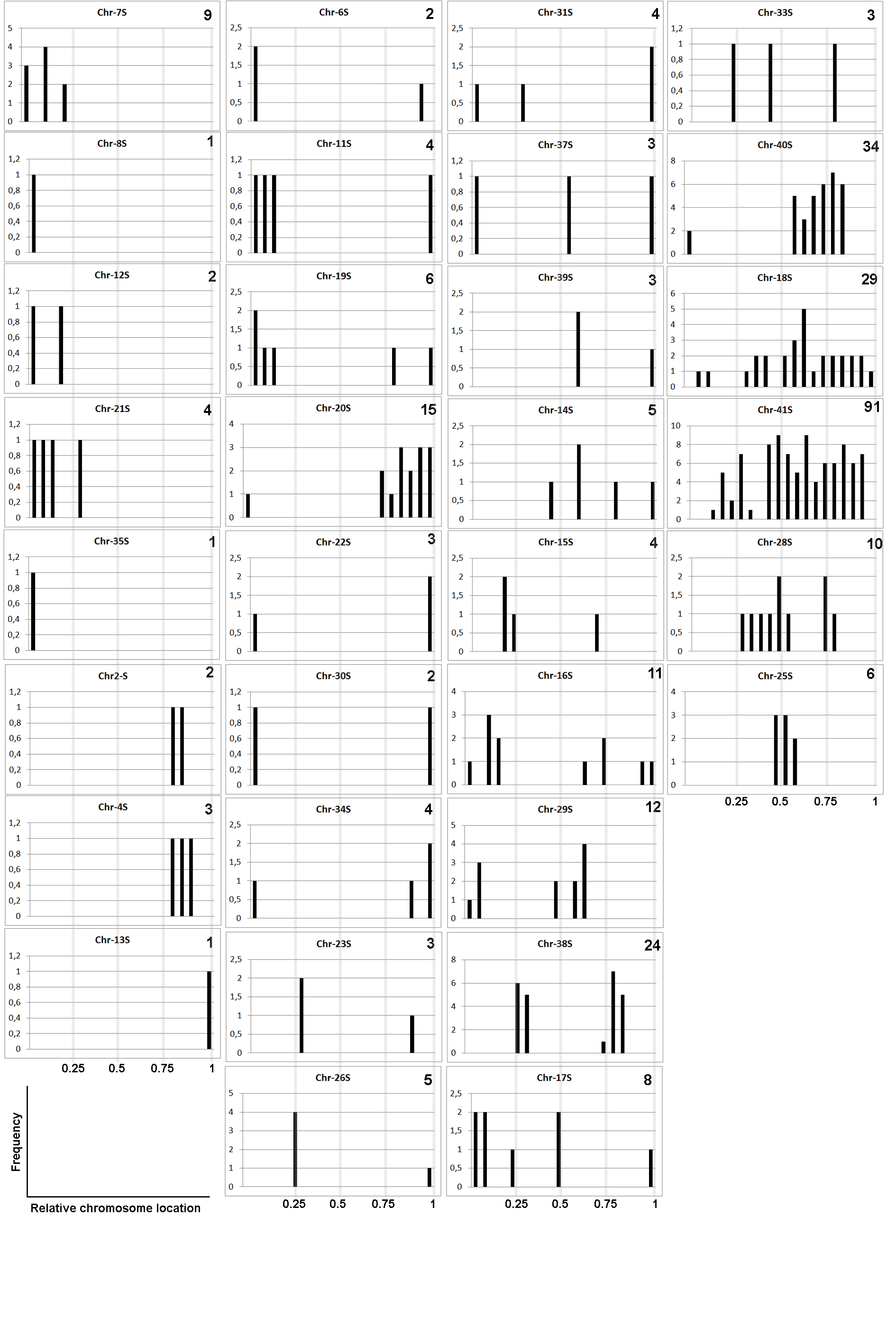

Supplement: Supplementary file 1 [file genes-11-01235-s001.zip › Supplementary File 5.jpg]

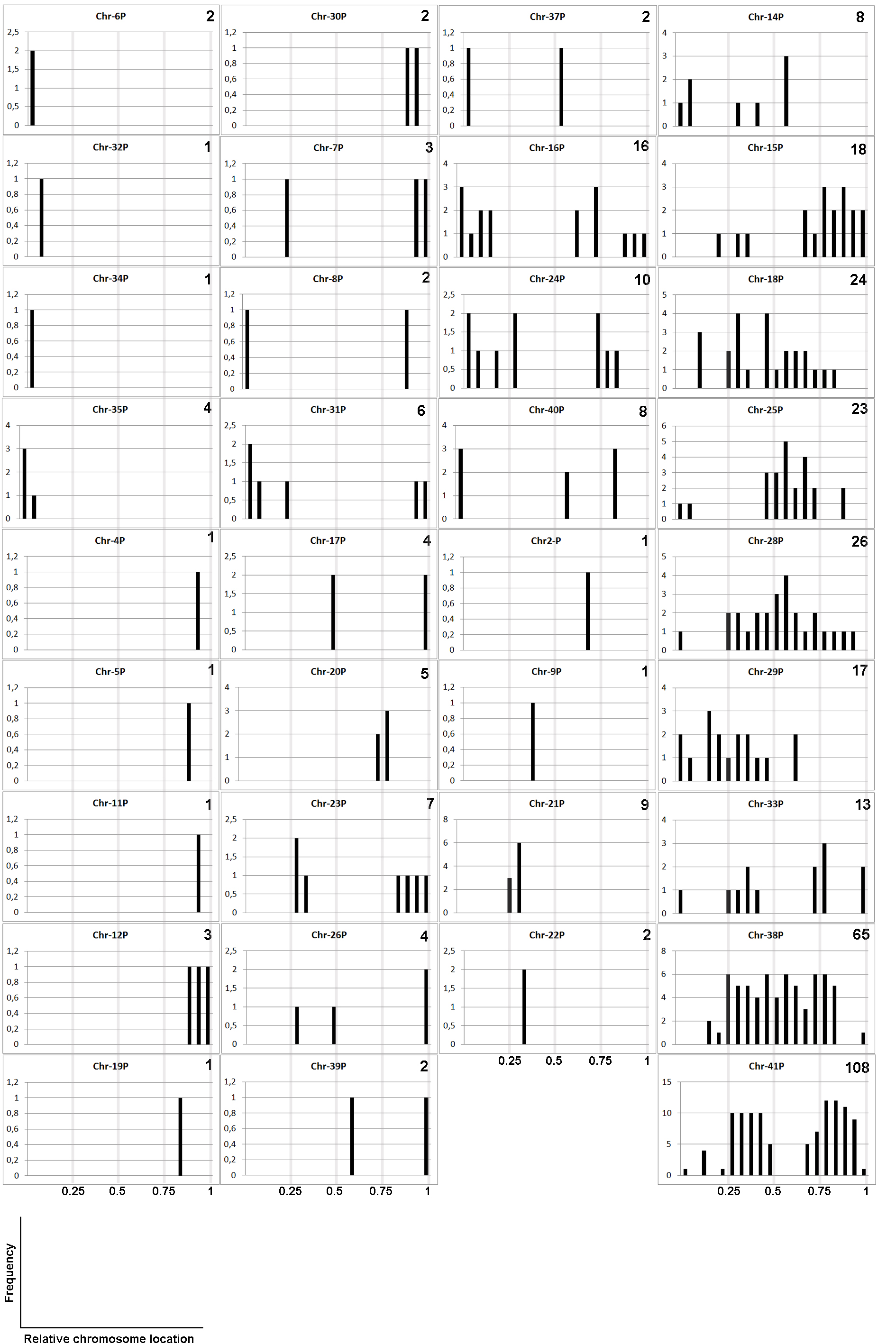

Supplement: Supplementary file 1 [file genes-11-01235-s001.zip › Supplementary File 6.jpg]

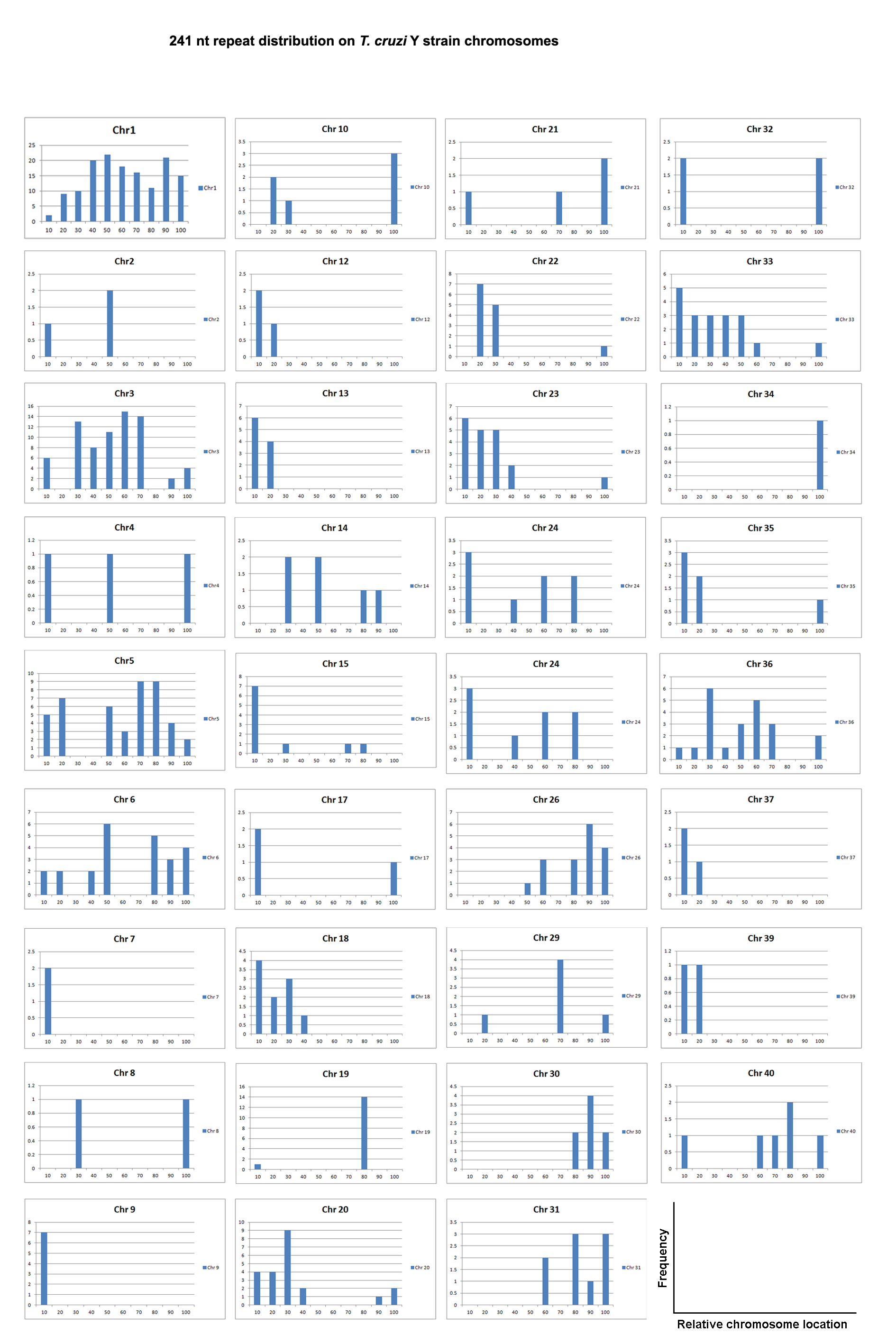

Supplement: Supplementary file 1 [file genes-11-01235-s001.zip › Supplementary File 7.jpg]

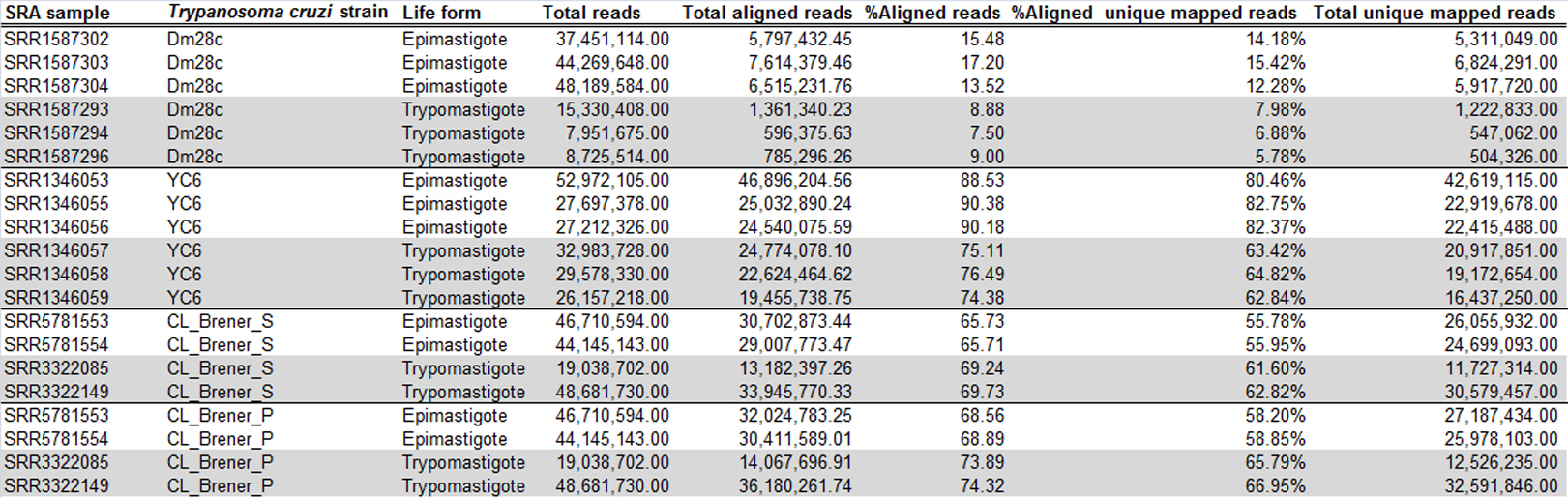

Supplement: Supplementary file 1 [file genes-11-01235-s001.zip › Supplementary File 10.jpg]
